# The skin microbiota of the axolotl *Ambystoma altamirani* is highly influenced by metamorphosis and seasonality but not by pathogen infection

**DOI:** 10.1186/s42523-022-00215-7

**Published:** 2022-12-12

**Authors:** Emanuel Martínez-Ugalde, Víctor Ávila-Akerberg, Tanya M. González Martínez, Montserrat Vázquez Trejo, Dalia Zavala Hernández, Sara Lucia Anaya-Morales, Eria A. Rebollar

**Affiliations:** 1 Centro de Ciencias Genómicas, Universidad Nacional Autónoma de México, Cuernavaca, Mexico; 2 Instituto de Ciencias Agropecuarias y Rurales, Universidad Autónoma del Estado de México, Toluca, Mexico; 3 Facultad de Ciencias, Universidad Nacional Autónoma de México, Mexico City, Mexico; 4 Department of Biology, University of Mississippi, Oxford, MS USA

**Keywords:** Skin microbiota, Amphibians, Metamorphosis, Seasonality

## Abstract

**Background:**

Microbiomes have been increasingly recognized as major contributors to host health and survival. In amphibians, bacterial members of the skin microbiota protect their hosts by inhibiting the growth of the fungal pathogen *Batrachochytrium dendrobatidis* (Bd). Even though several studies describe the influence of biotic and abiotic factors over the skin microbiota, it remains unclear how these symbiotic bacterial communities vary across time and development. This is particularly relevant for species that undergo metamorphosis as it has been shown that host physiology and ecology drastically influence diversity of the skin microbiome.

**Results:**

We found that the skin bacterial communities of the axolotl *A. altamirani* are largely influenced by the metamorphic status of the host and by seasonal variation of abiotic factors such as temperature, pH, dissolved oxygen and conductivity. Despite high Bd prevalence in these samples, the bacterial diversity of the skin microbiota did not differ between infected and non-infected axolotls, although relative abundance of particular bacteria were correlated with Bd infection intensity.

**Conclusions:**

Our work shows that metamorphosis is a crucial process that shapes skin bacterial communities and that axolotls under different developmental stages respond differently to environmental seasonal variations. Moreover, this study greatly contributes to a better understanding of the factors that shape amphibian skin microbiota, especially in a largely underexplored group like axolotls (Mexican *Ambystoma* species).

**Supplementary Information:**

The online version contains supplementary material available at 10.1186/s42523-022-00215-7.

## Background

Host associated microbiomes are vital for host health and survival, as they play relevant functions linked to nutrition, reproduction, behavior, defense against pathogens or predators [1–5]. Specifically, some animal associated microbiomes contribute to host health due to their ability to inhibit the growth of pathogens responsible for infectious diseases threatening diverse host species such as bats, snakes, or amphibians [6–8]. For instance, it has been shown that some members of the amphibian skin microbiome inhibit the growth of the lethal pathogens *Batrachochytrium dendrobatidis* (Bd) and *B. salamandrivorans* [9–12], which have caused amphibian populations declines and extinctions worldwide [13].

Studies accumulated over the past two decades showed that the amphibian skin microbiome is influenced by host associated factors (host genetics and development) [14–16], microhabitat related factors (environmental microorganisms, habitat abiotic conditions and pathogen presence) [17–21], and climatic and geographical factors (seasonality, precipitation, temperature or land use) [14, 22–25].

In the case of host-associated factors, it has been shown that the skin microbiota of amphibians (specifically frogs) changes across development and particularly before and after metamorphosis [26–28]. During metamorphosis amphibians in larval stages transition to adults following a series of physiological rearrangements such as tail reabsorption, limb development and remodeling of muscles, heart, intestine brain, and skin [29]. Metamorphosis also induces immunosuppression in response to thyroid and corticosteroid hormone signaling and eventually the immune system reorganizes and gradually matures in newly metamorphosed adults [30].

Along with physiological rearrangements, many amphibian species go through behavioral and lifestyle changes, while larval stages inhabit aquatic environments, adults become terrestrial and only return to water environments in the reproductive season [31–33]. These changes in microhabitat occupancy could influence skin microbial composition since the environmental microbial communities are one of the main sources of microbial diversity [16, 17].

In the case of climatic factors, temporal variation of abiotic factors [34] such as temperature and precipitation have a strong influence over amphibian skin microbial community structure [22, 35]. For example, in tropical regions microbial diversity on the amphibian skin differs between wet and dry seasons [19, 26, 36]. In temperate regions, where the four seasons are well defined through the year, seasonal changes have been linked to the temporal dynamics of the amphibian skin microbiota [22, 37–39]. In addition, it has been shown that spatial variation such as elevation gradients [40–42] or distinct microhabitats [43] influence the skin microbial diversity of amphibians.

Bd influence over the amphibian skin microbiota has been described in amphibian species with contrasting Bd infection status (infected–non-infected [19] and high Bd prevalence—low Bd prevalence [44]). These studies showed that disruption of skin microbiota following Bd infection can influence host survival and that the final outcome of the infection depends on the interplay between host, microbiome and the environment [21, 23, 45].

Here we analyzed the skin bacterial diversity of the axolotl *Ambystoma altamirani,* a stream dwelling salamander endemic to conifer and oak-pine forest from the central region of Mexico [46]. *A. altamirani* is a facultative paedomorphic species in which, metamorphic (without gills) and pre-metamorphic (with gills) individuals inhabit the same streams all year long [47, 48], allowing us to evaluate how metamorphosis and seasonality influence the skin microbiota in a species living in the same aquatic environment across time and development. In addition, we evaluated if skin microbiota differs from environmental bacterial communities and if Bd presence and infection intensity influence the skin microbiota of *A. altamirani*. We hypothesized that *A. altamirani* skin microbiota would (a) differ from environmental bacterial communities, (b) vary between metamorphic and pre-metamorphic salamanders, (c) change across seasons and (d) differ according to Bd infection status.

## Results

We sampled a total of 279 *A. altamirani* individuals (85 metamorphic and 194 pre-metamorphic) at four locations across four seasons. Additionally, 159 environmental samples from sediment (80) and water (79) were collected. After quality control and rarefaction at 10,000 reads per sample, 13 samples were discarded and the remaining 438 samples were used to perform all diversity analyses (Table 1). A final table with a total of 72,408 amplicon sequence variants (ASVs) was obtained including all samples.

**Table 1:** Final list of collected samples that passed bioinformatic filters

	Metamorphic	Pre-metamorphic	Sediment	Water	Total samples (N)
Summer (July 2019)	25	41	19	20	**105**
Autumn (October 2019)	28	29	20	20	**97**
Winter (January 2020)	9	66	20	20	**115**
Spring (April 2020)	23	58	20	20	**121**
Total samples (N)	**85**	**194**	**79**	**80**	**438**

Numbers in bold indicate the total number of samples collected for each sample type or season

Number of samples from the skin of *A. altamirani* individuals (metamorphic and pre-metamorphic) and environmental samples (sediment and water)

### *A. altamirani* skin microbiota differs from environmental bacterial communities

**Fig. 1:**
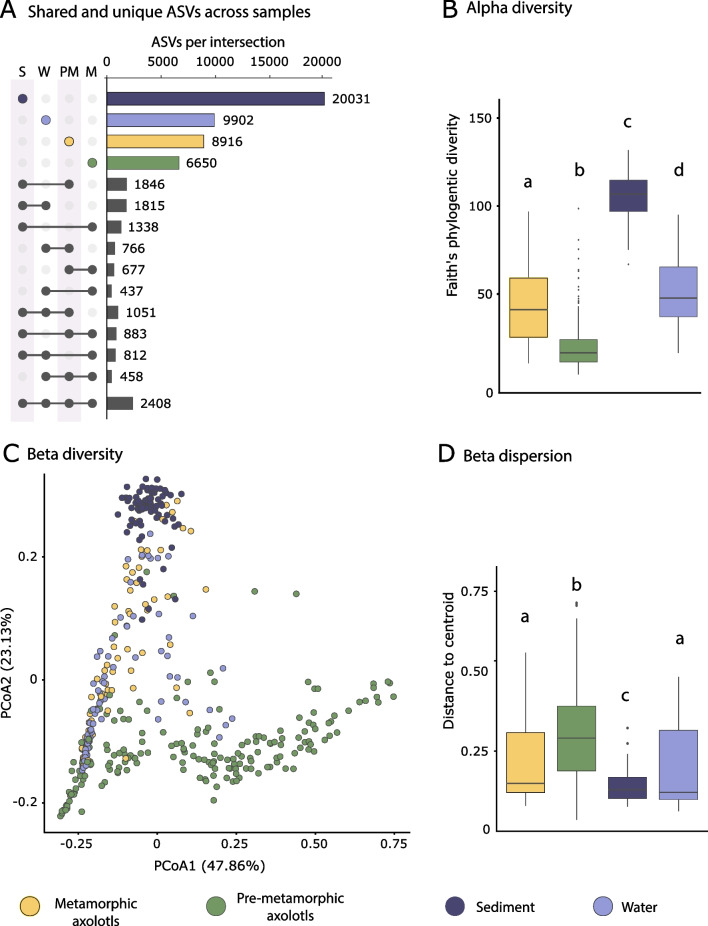
Bacterial diversity of *A. altamirani* skin and environmental samples. **A** Upset plot illustrating the number of unique and shared ASVs. Numbers aside the color bars indicate how many ASVs were present on each sample type (color bars) and shared between sample types (gray bars). **B** Alpha Faith’s Phylogenetic diversity (PD) across sample types. **C** Principal coordinate analysis (PCoA) based on weighted UniFrac distances across sample types. **D** Beta dispersion using Analysis of multivariate homogeneity of groups dispersions. Letters a–d indicate statistically significant comparisons

When comparing the number of unique and shared ASVs across sample types, we found that each sample type harbored many unique ASVs and only 2408 ASVs (3.32% of the total) were shared among the four sample types (Fig. 1A). Sediment and water samples were the samples with highest numbers of unique ASVs (20,031 and 9902 respectively), while metamorphic and pre-metamorphic samples had 8916, and 6650 unique ASVs respectively. Interestingly only 677 ASVs (1.16% of the total ASVs) were shared between metamorphic and pre-metamorphic salamanders.

Taxonomic results showed that, Burkholderiaceae was the most abundant bacterial family in all four sample types accounting for 32.6% and 51.1% of the relative abundance in metamorphic and pre-metamorphic samples respectively, and 14.6% and 40.8% of sediment and water respectively (Additional file 1: Figure S1). For the axolotl samples we found that Chitinophagaceae and Pseudomonadaceae varied in relative abundance according to host metamorphic status, with Chitinophagaceae showing a higher abundance in pre-metamorphic axolotls (metamorphic 2.7%/pre-metamorphic 27%) and Pseudomonadaceae being more abundant in metamorphic samples (metamorphic 18.1%/pre-metamorphic 6.4%).

Bacterial alpha diversity was significantly different between sample types (metamorphic, pre-metamorphic, sediment and water) as measured by ASV richness (Kruskal–Wallis (KW), $\chi 2$ = 278.46, *p*-value < 0.001, df = 3), Shannon index (KW, $\chi 2$ = 276.28, *p*-value < 0.001, df = 3) and Faith´s phylogenetic diversity (PD) (KW, $\chi 2$ = 286.91, *p*-value < 0.001, df = 3) (Fig. 1B). Post hoc pairwise comparisons for each alpha diversity index showed significant differences among all sample types (Additional file 2: Table S1) except for metamorphic salamanders and water in ASV richness (Wilcoxon, *p*-value = 0.48) and Shannon diversity index (Wilcoxon, *p*-value = 0.66). Sediment samples showed the highest alpha diversity values while pre-metamorphic salamanders always had the lowest values.

Bacterial community composition based on the weighted UniFrac distance matrix varied significantly among sample types (PERMANOVA, pseudo-F = 64.76, *p*-value < 0.001, df = 3) (Fig. 1C, Additional file 2: Table S2). Dispersion significantly differed among sample types according to the permutational test (PERMPUTEST, F = 34.5, *p*-value = 0.001, df = 3) (Fig. 1D, Additional file 2: Table S3).

### The skin bacterial composition of *A. altamirani* is mainly influenced by metamorphosis

**Fig. 2:**
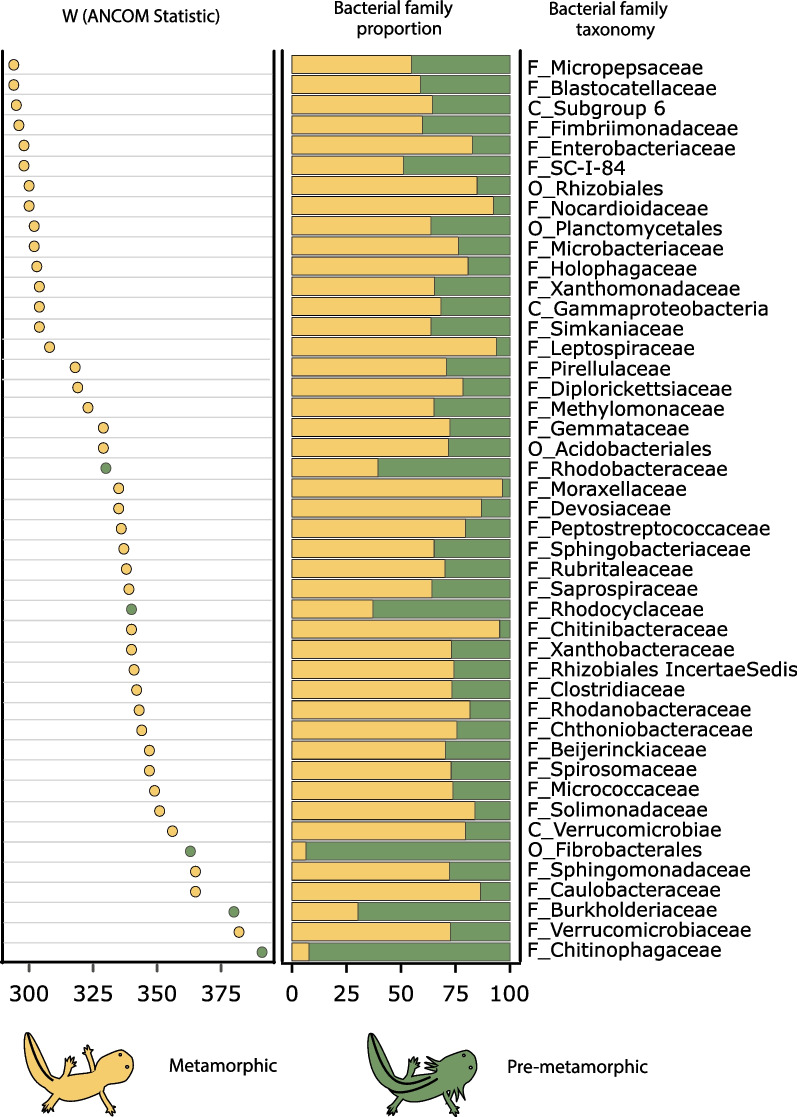
ANCOM results showing differentially abundant bacterial families between metamorphic and pre-metamorphic axolotls. Left panel shows ANCOM W values, the middle panel shows the relative proportion for each bacterial family, and the right panel shows the best taxonomic assignments according to the SILVA database at order (O), class (C) or family (F) level. Circles and bars are color-coded according to the host metamorphic status

Clear differences in skin bacterial alpha and beta diversity were found between metamorphic and pre-metamorphic salamanders (Fig. 1B, C, D). To look deeper into the bacterial taxa driving these differences we used an analysis of composition of microbiomes (ANCOM) which identified 45 bacterial families (out of 392 families in the axolotl skin samples) that were differentially abundant between metamorphic and pre-metamorphic samples (Fig. 2). Most of these bacterial families (40 out of 45) were enriched in metamorphic samples, being Verrucomicrobiaceae, Caulobacteraceae and Sphingomonadaceae the families with higher W values. In contrast, five bacterial families were enriched in pre-metamorphic samples with Burkholderiaceae, Chitinophagaceae being the families with higher W values.

We identified the core skin bacteria in metamorphic and pre-metamorphic *A. altamirani* axolotls, as those ASVs shared in ≥ 90% of the samples on each specific morph. Four ASVs represented the bacterial core of metamorphic axolotls accounting for a cumulative relative abundance of 16.26% of the ASVs. Meanwhile, two ASVs represented the bacterial core of pre-metamorphic axolotls accounting for 45.78% of the relative abundance (Table 2).

**Table 2:** Amplicon sequence variants (ASVs) defining the bacterial core of the microbiota of metamorphic and pre-metamorphic *A. altamirani*

	ASV ID	Taxonomy at family level	Relative abundance	Persistence
Metamorphic	9936daae333af6e517a9deb4b9e18ffa	Pseudomonadaceae	13.81	95.12
	6d0c9d0395e6a2a7667eb0b07c17a275	Burkholderiaceae	1.40	97.56
	17d60505100c3cf44d4f9fad620d1636	Pseudomonadaceae	0.73	93.90
	be8eb25874b4202cf98050dbadeeb7ce	Burkholderiaceae	0.33	93.90
Pre-metamorphic	3c28f0caf9183357de05d1882a943f8e	Chitinophagaceae	25.03	96.84
	ed5a79897d0f82525c3854759d384c26	Burkholderiaceae	20.75	98.42

ASVs were considered part of the skin bacterial core if they were present in ≥ 90% of the samples of metamorphic or pre-metamorphic axolotls

We also identified the core bacteria of environmental samples (Additional file 2: Table S4) and we found that metamorphic axolotls shared two core ASVs with the water core and another one with the sediment core. Core bacteria of pre-metamorphic axolotls are not present in the core of environmental samples.

### Seasonality and location differentially influence skin bacterial diversity in metamorphic and pre-metamorphic axolotls

Physicochemical variables measured at each sampling location (pH, conductivity, dissolved oxygen, maximin, minimum mean and delta seasonal temperatures) varied significantly across seasons (MANOVA, Wilks = 0.002, *p*-value < 0.001, df = 3) and sampling locations (MANOVA, Wilks = 0.0009, *p*-value < 0.001, df = 3). While all physicochemical variables varied across seasons, dissolved oxygen was the only variable that did not vary between sampling locations (Additional file 2: Table S5).

**Fig. 3:**
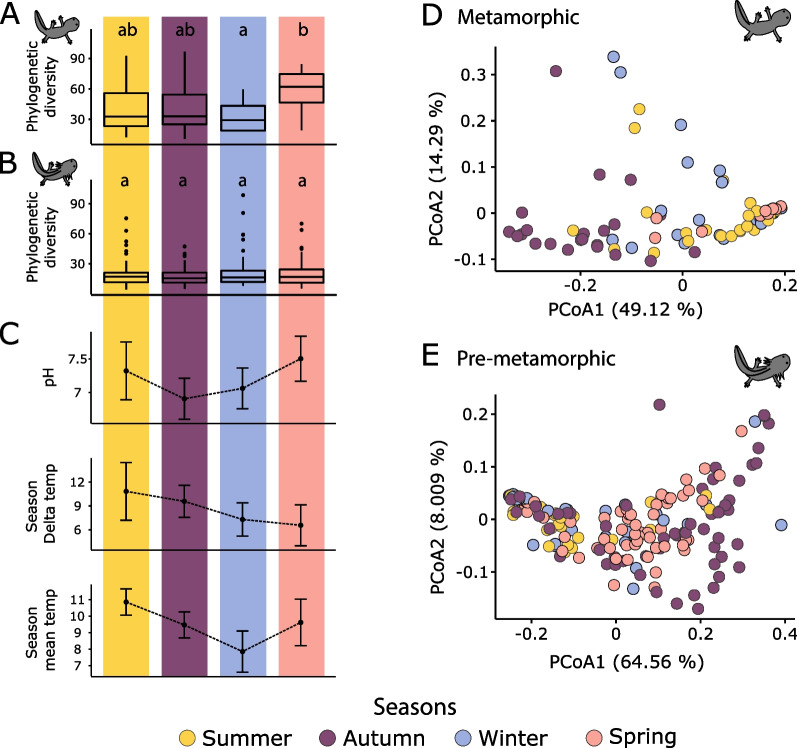
Seasonal influence over metamorphic and pre-metamorphic skin bacterial diversity. **A** Phylogenetic diversity (PD) across seasons in metamorphic samples. Letters a–d indicate statistically significant comparisons. **B** PD across seasons in pre-metamorphic samples. **C** Seasonal variation of pH, delta temperature and mean temperature of the stream water. **D** Principal coordinate analysis (PCoA) based on weighted UniFrac distances across seasons of metamorphic samples. **E** PCoA based on weighted UniFrac distances across seasons in pre-metamorphic samples. Circles in **D** and **E** panels are color-coded by season

Alpha PD of metamorphic axolotls varied significantly across seasons (KW, $\chi 2$ = 13.69, *p*-value = 0.003, df = 3) (Fig. 3A) and post-hoc pairwise comparisons showed that only the transition between winter-spring was significant (Wilcoxon, *p*-value = 0.005) (Additional file 2: Table S6). In contrast, PD of pre-metamorphic *A. altamirani* (Fig. 3B) did not differ across consecutive seasons (KW, $\chi 2$ = 0.21, *p*-value = 0.97, df = 3) (Additional fiie 2: Table S6).

Additionally, we found that seasonality significantly influenced skin bacterial community composition (PERMANOVA, pseudo-F = 12.37, *p*-value < 0.001, df = 3) (Fig. 3D, Additional file 2: Table S7), but not dispersion (PERMUTEST, F = 1.4, *p*-value = 0.24, df = 3) (Additional file 2: Table S8) of metamorphic axolotls. Seasonality also influenced skin bacterial community composition (PERMANOVA, pseudo-F = 15.69, *p*-value < 0.001, df = 3) (Fig. 3E, Additional file 2: Table S9) of pre-metamorphic axolotls, in addition we found that dispersion significantly differed between seasons (BETADISPER, F = 2.7, *p*-value = 0.038, df = 3) (Additional file 2: Table S10). Specifically, pairwise PERMANOVAs showed that metamorphic samples differed between winter-spring seasons (PERMANOVA, pseudo-F = 14.92, *p*-value = 0.001, df = 1), while pre-metamorphic skin microbiota differed between autumn–winter (PERMANOVA, pseudo-F = 13.47, *p*-value < 0.001, df = 1) and winter-spring seasons (PERMANOVA, pseudo-F = 12.61, *p*-value < 0.001, df = 1).

**Fig. 4:**
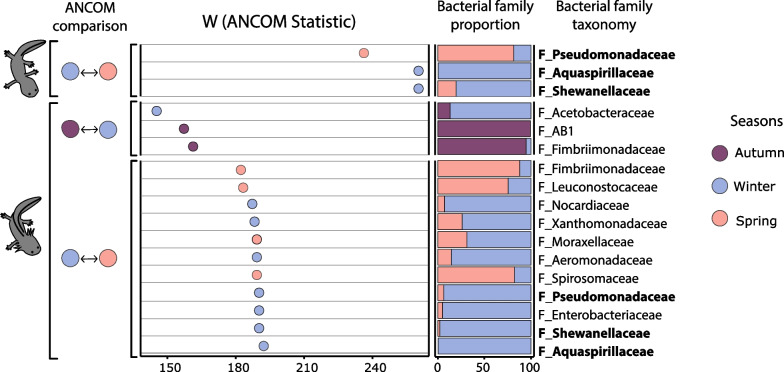
ANCOM results showing differentially abundant bacterial families in metamorphic and pre-metamorphic axolotls across consecutive seasons: autumn to winter seasons in pre-metamorphic axolotls, and winter to spring seasons for metamorphic and pre-metamorphic axolotls. From left to right: ANCOM comparisons color-coded by season, ANCOM W values, the relative bacterial family proportion and the best taxonomic assignment according to SILVA at order (O), class (C) or family (F) level. Circles and bars are color-coded by season. Shared bacterial families between metamorphic and pre-metamorphic axolotls between winter and spring seasons are shown in bold

Three bacterial families were identified by ANCOM as differentially abundant in metamorphic samples between winter-spring seasons (Fig. 4). In the case of pre-metamorphic individuals, ANCOM identified three bacterial families that were differentially abundant between autumn–winter and eleven families differentially abundant between winter-spring (Fig. 4). Pseudomonadaceae, Aquaspirillaceae and Shewanellaceae were significantly enriched in both metamorphic and pre-metamorphic axolotls during winter and spring seasons. However, Pseudomonadaceae was more abundant in metamorphic axolotls during spring and more abundant in pre-metamorphic axolotls during winter. Shewanellaceae was more abundant in winter, and Aquaspirillaceae was present in the winter and completely absent in the spring for both metamorphic and pre-metamorphic axolotls.

When analyzing the effect of location in the skin bacterial diversity, we found that PD of metamorphic samples differed significantly between sampling locations (KW, $\chi 2$ = 9.69, *p*-value = 0.02, df = 3), however post hoc paired test showed that PD only differed significantly between sites 2 and 3 (Additional file 1: Figure S2A). Bacterial PD of pre-metamorphic samples also varied significantly between sampling locations (KW, $\chi 2$ = 40.9, *p*-value = 6.71e-9, df = 3). Post hoc test showed that most pairwise comparisons were significant with the exception of sites 1 and 3 and sites 2 and 3 (Additional file 1: Figure S2C, Additional file 2: Table S11). Skin bacterial community composition was also influenced by sampling location in metamorphic (PERMANOVA, pseudo-F = 2.71, *p*-value = 0.006, df = 3) and pre-metamorphic samples (PERMANOVA, pseudo-F = 31.34, *p*-value = 0.001, df = 3) (Additional file 1: Figure S2B, D). Pairwise comparisons showed that bacterial community composition only differed between sites 2 and 3 in metamorphic axolotls (Additional file 2: Table S12), while community composition differed between all sampling locations for pre-metamorphic samples (Additional file 2: Table S13). Interestingly dispersion did not vary across localities for metamorphic axolotls (PERMUTEST, F = 0.29, *p*-value = 0.8, df = 3) (Additional file 2: Table S14), but we found significant differences in dispersion for pre-metamorphic axolotls (PERMUTEST, F = 6.68, *p*-value = 0.01, df = 3) (Additional file 2: Table S15).

### Biotic and abiotic factors influence the skin bacterial community structure of *A. altamirani*

Our results showed that bacterial community composition of *A. altamirani* skin is influenced by seasonality and location. To assess the specific influence of all the biotic and abiotic factors measured in this study we performed a distance-based Redundancy Analysis (dbRDA) on the skin bacterial beta diversity. After forward model selection, that incorporates all the variables measured, only the biotic and abiotic factors that better explained community composition were included in the dbRDA regression model: host metamorphic status, host weight, pH, dissolved oxygen, conductivity, mean temperature, season delta temperature (difference between the maximum and minimum seasonal temperature) and site elevation.

**Fig. 5:**
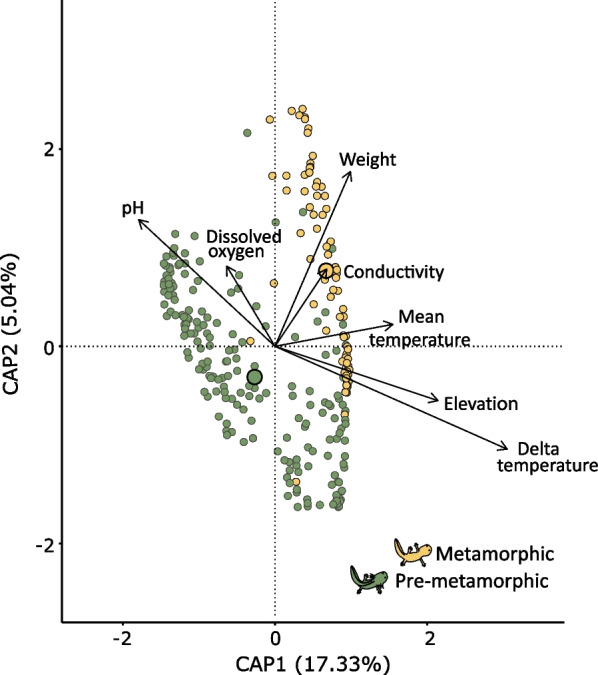
Distance based redundancy analysis of *A. altamirani* skin bacterial communities. Distances in the PCA are based on a weighted UniFrac distance matrix. Vector directions indicate the type of correlation of each predictor variable. Distance of each sample with respect to vectors highlight the weight of the correlation with a given predictor variable. Non quantitative variables are represented as centroids (outlined circles larger). Circles are color-coded by host metamorphic status

The dbRDA calculated eight canonical components for the PCA, however anova.cca (by = axis) showed that only four of these canonical components were statistically significant. These four statistically significant canonical axes explained 26.47% of the variation in the weighted UniFrac distance matrix (Fig. 5, Additional file 2: Table S16). Permutational analysis (anova.cca, by = terms) over each variable in the model showed that the metamorphic status of the host (PERMANOVA, pseudo-F = 39.1, *p*-value = 0.001) had the greatest effect-size over the variation, followed by seasonal delta temperature (PERMANOVA, pseudo-F = 19.8, *p*-value = 0.001), pH (PERMANOVA, pseudo-F = 15.85, *p*-value = 0.001) and seasonal mean temperature (PERMANOVA, pseudo-F = 12.05, *p*-value = 0.001) (Fig. 3C, Table 3).

**Table 3:** Permutational like ANOVA results of each variable introduced in the dbRDA regression model

	F	*p*-value
Developmental stage	39.121	**0.001**
Delta temperature	19.889	**0.001**
pH	15.854	**0.001**
Mean temperature	12.053	**0.001**
Elevation	5.334	**0.001**
Dissolved oxygen	4.478	**0.002**
Conductivity	3.470	**0.005**
Weight	2.604	**0.018**

Columns indicate: F statistic, p-values calculated by Permutational like ANOVA for each variable

Numbers in bold indicate significant *p*-value

### Skin bacterial diversity of *A. altamirani* is not influenced by Bd infection status but specific bacterial taxa abundances correlate with infection intensity

Pathogen prevalence and infection intensity were conducted by Basanta et al. [49]. Briefly we found a Bd prevalence of 70.3% across all samples specifically 54 (out of 85) metamorphic and 142 (out of 194) pre-metamorphic axolotls resulted positive for Bd infection.

**Fig. 6:**
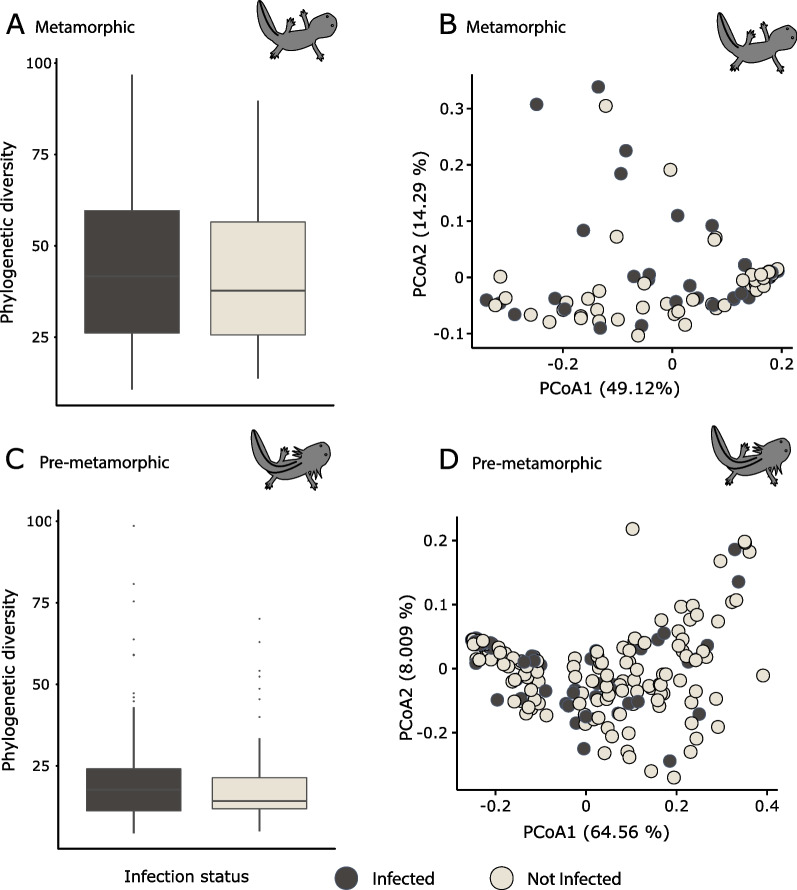
*A. altamirani* skin bacterial diversity with respect to Bd infection status. **A** Alpha phylogenetic diversity (PD) between infected and non-infected in metamorphic axolotls. **B** Principal coordinate analysis (PCoA) based on weighted UniFrac distances in infected vs non-infected metamorphic axolotls. **C** PD between infected and non-infected in pre-metamorphic axolotls. **D** PCoA based on weighted UniFrac distances in infected vs non-infected in pre-metamorphic axolotls. Circles are color-coded by Bd infection status

Alpha PD did not differ between infected and non-infected samples in both metamorphic (KW, $\chi 2$ = 0.09, *p*-value = 0.76, df = 1) (Fig. 6A) and pre-metamorphic (KW, $\chi 2$ = 0.51, *p*-value = 0.47, df = 1) *A. altamirani* samples (Fig. 6C). Additionally, beta diversity based on the weighted UniFrac distance matrix did not vary between infected and non-infected samples for metamorphic (PERMANOVA, pseudo-F = 1.37, *p*-value = 0.19, df = 1) (Fig. 6B) and pre-metamorphic axolotls (PERMANOVA, pseudo-F = 2.45, *p*-value = 0.08, df = 1) (Fig. 6D).

Even though alpha and beta diversity did not vary according to Bd infection status, Kendall’s correlation test showed that the relative abundance of 139 and 129 ASV present in infected metamorphic and pre-metamorphic samples respectively, significantly correlated with pathogen infection loads (Additional file 1: Figure S3). Specifically, 116 (out of 139) and 52 (out of 128) bacterial ASVs had positive correlations with pathogen infection loads in metamorphic and pre-metamorphic samples, respectively.

Almost all the ASVs that correlated with pathogen load in metamorphic samples had low relative abundances ranging from 0.001 to 0.67% (Additional file 1: Figure S3A), while in pre-metamorphic samples, correlated ASVs ranged from 0.001 to 28.5% (Additional file 1: Figure S3B). Among the ASVs that correlated with pathogen infection intensities, twelve of them were shared between metamorphic and pre-metamorphic axolotls and half of these ASVs had a differential type of correlation between morphs (e.g., positive in metamorphic and negative in pre-metamorphic) (Additional file 2: Table S17). Two of these ASVs were classified as members of the Chitinophagaceae family and both were positively correlated with Bd infection intensity in metamorphic axolotls and negatively correlated in pre-metamorphic axolotls.

## Discussion

The aim of this study was to evaluate the influence of metamorphosis, seasonality and pathogen presence over the skin microbiota of the axolotl *A. altamirani*. Since this is the first study exploring the skin microbiota of *A. altamirani*, we also evaluated if skin bacterial diversity differed from environmental bacterial communities of the streams where this species inhabits.

Consistent with previous studies showing differences between amphibian skin microbiota and their surrounding environmental bacterial communities [20, 50], we found that *A. altamirani* skin bacterial microbiota significantly differed from environmental samples, and that a great portion of the ASVs were unique to each sample type (ie, sediment, water, metamorphic and pre-metamorphic axolotls, Fig. 1A), supporting the idea that the amphibian skin hosts a distinctive bacterial repertoire compared to the environmental samples [18, 35, 37, 43, 51]. Our results highlight differences on the microbial diversity between the bacterial communities of the skin and the environment. We identified differences in alpha and beta diversity compared to water and sediment. Also, we identified core bacteria that were unique to axolotls or were clearly enriched on their skins compared to the environment.

Several studies have shown that amphibian skin microbiota varies significantly across host development [26, 27, 41]. These studies focused on amphibian species that transition from an aquatic larval stage to a terrestrial adult stage [22, 36, 37, 52], making it difficult to tease apart the effects of host development stage and habitat conditions on skin microbial diversity [17, 18]. For species where adult and larval stages coexist in the same aquatic environment (i.e., newts), host developmental stage has had contrasting results in different species; for example adult and larvae of *Lissotriton boscai* showed clear differences in skin bacterial community composition, however this pattern was not observed in *Triturus marmoratus* [52].

In this study, we evaluated the influence of metamorphosis over skin bacterial diversity on a paedomorphic salamander species (axolotl) in which metamorphic and pre-metamorphic stages coexist in permanent streams along their life cycle [47, 53]. Our results showed that *A. altamirani* skin bacterial communities are strongly shaped by metamorphosis. Specifically, we found that pre-metamorphic individuals harbor less diverse and more variable skin bacterial communities compared to metamorphic individuals.

These differences could be explained by differences in skin mucus composition, immune response, or gene expression before and after metamorphosis as it has been proposed that mucus chemical composition (e.g., production of antimicrobial peptides) play a critical role in shaping the skin microbiota as well as in defense against pathogens [30, 54, 55]. Antimicrobial peptide repertory of the skin changes through development in some frog species [56], and some bacteria can induce the synthesis of specific antimicrobial peptides [51]. In addition, the number and distribution of Leydig cells, which have been associated with the secretion of mucus [57], changes across urodele development [58, 59].

In addition, the core microbiota analysis and ANCOM results shown here highlighted differences in composition between metamorphic and pre-metamorphic axolotls. Specifically, pre-metamorphic skin microbiota was composed by fewer core members and had less differentially abundant bacterial ASVs when compared to metamorphic skin microbiota. It is interesting to highlight that both analyses identified that families Chitinophagaceae and Burkholderiaceae were enriched in pre-metamorphic samples, specially two ASVs from these families conform the core microbiota of pre-metamorphic axolotls which account 45.7% of relative abundance in these samples.

Bacteria from the family Chitinophagaceae and Burkholderiaceae have been isolated from other amphibian hosts and have shown the ability to inhibit Bd [60–62]. Moreover, some members of the Chitinophagaceae family such as *Chitinophaga pinenis* can degrade chitin [63] which is a main component of fungal cell wall. In our study, the high prevalence of these bacterial families on the skin of *A. altamirani* could suggest that these bacteria play a protective role against chytrid pathogens.

Temporal and spatial dynamics of amphibian skin microbiota have been linked to variation in environmental factors such as temperature, precipitation or elevation [25, 26, 34, 35, 52]. Specifically, temperature fluctuations over short periods of time [22] and seasonal variations (dry–wet) [38] have been linked to differences in bacterial skin diversity on amphibians inhabiting aquatic environments. Our results showed that seasonal variation of temperature (delta temperature and mean temperature), pH, conductivity, and dissolved oxygen influence axolotl skin bacterial diversity.

Previous studies have shown that spatial variation has an influence on skin bacterial diversity of terrestrial salamanders [14, 41, 64]; for example populations of *Ensatina eschscholtzii* from different geographic locations vary in bacterial community composition [15]. In this study we found that sampling location significantly influences skin bacterial diversity, and this effect is stronger in pre-metamorphic axolotls. Considering that the main source of diversity of the skin microbiota are the environmental microbial communities and that they vary in response to environmental variation [65–67] it is likely that the skin microbiota reflect to some extent the environmental variations across localities as seen in the case of pre-metamorphic axolotls. It has been shown that skin bacterial diversity vary in response to precipitation [19, 23] temperature [22] or elevation gradients [24, 41, 42]. However, genetic differences across populations could also explain some of our results, since a previous study showed that *A. altamirani* populations of sites 2 and 3 are genetically differentiated [68]. Additional work is needed to tease apart the effects of environment and host genetics on the skin microbial diversity of *A. altamirani*.

Stability as a characteristic of an ecological community could be defined as the response to disturbance, comprising resilience and resistance against external disturbances [69, 70]. It has been shown that the stability of the amphibian skin microbiota can change after the experimental exposure to fungal [71] and viral pathogens [72]. Also, it has been shown that skin microbiomes with higher diversity are less stable to a pathogen induced disturbance [72].

Environmental variation across seasons is a different kind of perturbation for microbial communities, and it has been shown that seasonality influences microbial communities of soil [73–75], water [76, 77] and host-associated microbiomes [78, 79]. We found that skin bacterial communities of *A. altamirani* vary across seasons, particularly in pre-metamorphic axolotls which have a lower bacterial diversity compared to metamorphic axolotls.

Together our results suggest that more diverse bacterial communities (as the ones present in metamorphic axolotls) allow for a more stable microbiota that could be either more resistant or resilient to the environmental variation. Similar patterns of diversity—stability trough time have been described in populations of *Rana sierrae* [39]. Further studies are needed to evaluate if these patterns of stability across seasons influence the function of the skin bacterial communities of *A. altamirani*, as it has been shown that less stable bacterial communities show less functional redundancy [80].

Disruption of the skin microbiota following Bd infections has been previously documented in naive amphibian populations before and after Bd infection [20, 21], and in populations with different pathogen intensities where Bd seems to be present in an enzootic stage [23, 44]. Even when Bd was highly prevalent in *A. altamirani* populations [49], we did not find any significant influence of Bd presence over the skin bacterial diversity.

Of the 279 axolotls sampled only two individuals exhibited clear signs of infection [81] (lethargy, skin ulceration and extreme skin sloughing) and they died soon after we sampled them. Apart from these two cases, the remaining individuals showed no signs of infection. These observations suggest that this population is able to tolerate Bd infection. Further studies testing the survival rates of *A. altamirani* against Bd are needed to elucidate if this species is resistant or susceptible to chytridiomycosis.

It has been shown that Bd presence have contrasting effects over skin microbiota diversity inducing changes in skin microbiota composition following infection [21, 23, 71] or not influencing diversity of skin microbial communities [42, 44] as we found in this study. However, it also has been shown that relative abundances of some bacterial members of the skin microbiota correlates with chytrid infection intensity [19, 44, 45] and its suggested that according to the type of correlation these groups could act as anti Bd bacteria [19]. We identified several bacteria with positive and negative correlations with Bd infection intensities and most of these ASVs exhibited low relative abundances.

Observations in several amphibian species indicate that certain bacteria with properties such as biofilm formation [82] or putative inhibitory ability [55] are positively or negatively corelated with a decrease of Bd prevalence. Thus, we expect that bacteria with negative correlations to infection intensity could be important in the defense against Bd in *A. altamirani*. However, these Bd-inhibitory bacteria exhibited reduced abundances over the amphibian skin [83, 84].

Inhibitory potential against Bd has been described for several bacterial isolates mainly form Burkholderiaceae, Yersiniceae, Pseudomonadaceae or Xanthomondaceae families [60, 85–88], We found that Burkholderiaceae and Chitinophagaceae were highly abundant over *A. altamirani* skin. In line with our results, high abundance of Burkholderiaceae in *Anaxyrus boreas* skin microbiota correlated with reduced fungal presence over the skin during early life stages [27]. Additionally, populations of *R. sierrae* with contrasting Bd loads (high vs low) exhibited differential abundances of Burkholderiaceae [21, 44]. In the case of Chitinophagaceae little is known about their inhibitory ability against Bd with only few isolates considered as Bd-inhibitory strains [25], and further work is needed to elucidate if members of this bacterial family present on *A. altamirani* skin display inhibitory functions against Bd.

## Conclusion

Our results show that host metamorphic status is a major determinant of *A. altamirani*, influencing diversity and structure of skin symbiotic bacterial communities. To our knowledge this study is the first to address how the effects of environmental variation over the skin bacterial communities are dependent on the amphibian developmental stage; we demonstrate that seasonal environmental variation significantly influences bacterial skin diversity of *A. altamirani*, and that metamorphic and pre-metamorphic axolotls respond differently to environmental variation. Despite a growing body of literature suggesting that Bd influences skin bacterial diversity we did not find such an effect. Nonetheless, we found that particular bacterial taxa are likely interacting with Bd. Further studies using metagenomics and cultivation techniques could elucidate if changes in skin microbiota across development and across seasons are reflecting functional differences regarding Bd inhibition or other host symbiotic traits [89, 90].

## Methods

### Sample collection

Skin samples were collected during four sampling periods at three-month intervals (July 2019, October 2019, January 2020, and April 2020) spanning all the seasons of a whole year at four localities at La Sierra de Cruces, Estado de México, México (Table 1, Additional file 2: Table S18). Individuals of *A. altamirani* were captured at each location using dip nets and held individually in sterile plastic containers filled with stream water until swabbing. Sampling occurred for three consecutive hours across a 150 m transect along each stream. Each captured salamander was manipulated with sterile nitrile gloves, rinsed with 25 ml of sterile deionized water to remove transient microorganisms from the skin and swabbed 30 times (five times in their ventral and dorsal surface each and five times in each limb joint) using sterile rayon swabs (MWE, Corsham UK). Swabs were stored in 1.5 ml microcentrifuge tubes containing 170 μl of DNA/RNA Shield (Zymo Research, Irvine, USA) and kept at 4 °C during field work. Once in the laboratory tubes were stored at − 80 °C until processing. Immediately after swabbing morphometric measurements of weight, tail and body length were registered for each individual. Once all axolotls were swabbed and measured, they were released at the same site of capture. Sampling was approved by Subsecretaría de Gestión para la Protección Ambiental under the permit number: SGPA/DGVS/5673/19.

For the purposes of this work, we classified axolotl samples as metamorphic and pre-metamorphic according to the presence or absence of gills as reported previously [59]. Recognizing that gilled individuals of *A. altamirani* could be either juvenile or paedomorphic adults, we classified non-gilled axolotls as metamorphic and gilled axolotls as pre-metamorphic respectively in order to evaluate the effect of the metamorphic status of the host.

Additionally, five samples of sediment and water were collected at each location in all sampling periods. Water samples were obtained by submerging a sterile rayon swab at approximately 20 cm deep inside water for 10 s, and sediment samples were obtained by submerging swabs inside the bottom sediment of the stream for 10 s [50].

### Environmental characterization

Stream water temperature was recorded at 1 h intervals during one year at each sampling location using Onset HOBO dataloggers (Onset Computer Corporation, Bourne, USA) from June 2019 to April 2020. Additionally, pH, dissolved oxygen and conductivity of the water was registered using a HANNA multiparameter HI98194 (HANNA Instruments, USA) during each sampling. Measurements were taken at each location in triplicate across 10 m transects. To evaluate if these physicochemical variables vary between seasons and sampling location, we applied a two-way MANOVA test in R (v 4.0.2).

### DNA extraction and sequencing

Amplicon libraries of the 16S rRNA gene spanning the V4 region were constructed using 515F/806R primers following the Earth Microbiome Project standard protocol (www.earthmicrobiome.org) and previously published studies [50, 91]. In brief, DNA was extracted from skin and environmental swabs using the Qiagen DNeasy Blood and Tissue kit (Qiagen, Valencia, USA) following manufacturer instructions with an initial lysozyme incubation step at 37° for 1 h. Samples were PCR amplified in triplicate plus one negative control per sample, PCR products and negative controls were verified in 1% agarose gels, and PCR products were pooled in one tube per sample. Pools were quantified using a Qubit 4.0 fluorometer (Invitrogen, Thermo Fisher Scientific, Waltham, USA), samples were pooled in two amplicon libraries at a concentration of 240 ng per sample (221 and 217 samples each). Each pool was cleaned using the QIAquick PCR clean up kit (Qiagen, Valencia, USA). 16S amplicon libraries were sequenced in two sequencing runs (250 single end) using v2 Illumina chemistry at Dana-Farber Cancer Institute of Harvard University.

### Bioinformatic pipeline

Sequences were processed using Quantitative Insights Into Microbial Ecology (QIIME v2-2020.2) [92]. A total of 8,434,775 and 8,821,621 demultiplexed raw sequences were obtained from the sequencing facility for each sequencing run respectively. Prior to quality control primers were trimmed from the sequences using the cut-adapt plugin in Qiime2 then sequences were quality filtered and denoised independently for each run using the DADA2 plugin to obtain two single feature table. Feature tables obtained for each sequencing run were merged to generate a final Amplicon Sequence Variant (ASV) table containing 14,415,727 reads with a mean read depth of 32,900 reads per sample.

A phylogenetic tree was generated using the representative sequences of the ASV table using the q2-phylogeny plugin which first uses mafft to perform sequence alignment and then generate a phylogeny using FastTree. Samples were rarefied at 10,000 reads per sample according to observed ASV rarefaction curves in order to preserve the largest number of samples and sequences. After denoising and rarefaction at 10,000 reads per sample, seven axolotl (out of 279, three from metamorphic and four from pre-metamorphic) and eight environmental (out of 159, 3 from sediment and five from water) samples were discarded due to low read counts (< 10,000 reads per sample). The rarefied table containing 10,000 reads per sample was used for all further analyses including to calculate alpha and beta diversity metrics using the q2-diversity plugin. Taxonomy was assigned using a naive Bayesian classifier pre-trained for the V4 region (515F/806R 16 s rRNA) on the SILVA 132 99% database [93].

### Microbial diversity and composition analyses

Statistical analyses for alpha and beta diversity were carried out using the rarefied table at 10,000 sequences per sample; these analyses were computed in R (v 4.0.2) unless otherwise stated. We first perform Kruskal–Wallis (KW) and post hoc Wilcoxon ranks sum test were used to determine differences in alpha diversity (Shannon, Faith’s Phylogenetic Diversity (PD) and ASV richness) between sample types (metamorphic, pre-metamorphic, sediment, and water. In addition we perform KW and post hoc Wilcoxon ranks sum test to evaluate the influence of seasonality, sampling location and Bd infection status over the skin microbiota of metamorphic and pre-metamorphic axolotls individually.

Beta diversity was evaluated using a weighted UniFrac distance matrix generated using the rarefied table at 10,000 sequences per sample to determine differences in bacterial community structure across sample types. In addition, we generated two independent weighted UniFrac distance matrices for metamorphic and pre-metamorphic axolotls to evaluate the influence of seasonality, sampling location and Bd infection status. Statistical comparisons were conducted with permutational multivariate analyses (PERMANOVA) using the q2-diversity plugin in Qiime2 (v 2020.2). Beta diversity dispersion was calculated from the each weighted UniFrac distance matrix using the function betadisper in the vegan package [94], and then we applied PERMUTEST based on 999 permutations to identify significant differences for dispersion, specifically we evaluate dispersion between sample types, as well between seasonality and sampling locations for the metamorphic and pre-metamorphic distance matrices.

ANCOM [95] was used to identify bacterial families that were differentially abundant between metamorphic and pre-metamorphic salamanders and between samples from consecutive seasons (summer-autumn, autumn–winter, winter-spring). Prior to analysis low abundant ASVs (< 50 reads) were filtered out and then we collapsed all ASVs at family level, ANCOM was performed using the q2-composition plugin in Qiime2. Briefly, ANCOM applies a centered log ratio transformation on the relative abundance of each bacterial family and tests the null hypothesis that mean log absolute abundance of each family does not differ between sample types. An internal statistic (W) is calculated each time a taxon rejects this null hypothesis, then ANCOM generates an empirical distribution using W values in order to test which taxon in this case which bacterial families are differentially abundant between samples. ANCOM between consecutive seasons was only applied if PERMANOVA results showed significant differences between consecutive seasons (winter-spring for metamorphic salamanders and autumn–winter and winter-spring for pre-metamorphic salamanders).

Core microbiome was calculated independently for metamorphic and pre-metamorphic axolotls using the feature-table plugin in Qiime2. In brief, we generated four feature tables that contain all the ASVs present in each sample type (metamorphic, pre-metamorphic, sediment and water samples). Then we identify all the ASVs present in ≥ 90% of the samples of each sample type, using the core-features function.

Additionally, correlations between the relative abundance of each ASV of the infected samples and Bd infection intensities were calculated with Kendall rank correlation coefficient correcting for multiple comparisons (Benjamini-Hochberg) using cor.test function of the stats package in R [96]. To generate graphics for all the results Qiime2 artifacts were imported to R using the package qiime2R [97], then figures were generated using packages ggplot2 [98, 99], Fantaxtic [100] and UpSetR [101], color pallet of the figures are colorblind friendly and were selected from the MetBrewer package in R [102].

### Biotic and abiotic factors influencing the skin microbial structure

In order to explore the specific influence of biotic (developmental stage, weight, tail length, snout vent length, Bd presence and Bd infection intensity) and abiotic factors (pH, conductivity, dissolved oxygen, mean season temperature, delta season temperature, and elevation) on skin bacterial community composition, we applied a distance-based redundancy analysis (dbRDA) on the weighted UniFrac distance matrix using the capscale function of the vegan package [94]. dbRDA is a canonical ordination method that applies multiple linear regression to a distance matrix and then computes a principal component analysis (PCA) [103]. Prior to analyses non-categorical biotic and abiotic variables were z-scored to control for differences in magnitudes between factors. The ordistep function of the vegan package [94] was used for model selection in both directions with 999 permutations to select the best regression model. Once the dbRDa was obtained anova.cca function was used to perform an ANOVA like permutation test to evaluate the significance of each calculated canonical axis (anova.cca, by = axix) and the specific significance of each factor in the regression model (anova.cca, by = terms).

## Supplementary Information

**Additional file 1.** Supplementary Figures.

**Additional file 2.** Supplementary Tables.

## Data Availability

All 16 s rRNA gene raw data in this study are publicly available at the NCBI SRA under BioProject PRJNA819099. Sample metadata, output data of DADA2, R and Qiime2 scripts for analysis and figures included in this manuscript are available at https://github.com/EmanuelMartinez-Ugalde/A.-altamirani-16S-skin-microbiota.

## Abbreviations

ANCOM: Analysis of composition of microbiomes
ASV: Amplicon sequence variant
Bd: *Batrachochytrium dendrobatidis*
KW: Kruskal–Wallis
dbRDA: Distance based redundancy analysis
MANOVA: Multivariate analysis of variance
PD: Phylogenetic diversity
PERMANOVA: Permutational multivariate analysis of variance
PERMUTEST: Permutation-based test of multivariate homogeneity of group dispersions
