# Supplementary material for: The skin microbiota of the axolotl Ambystoma altamirani is highly influenced by metamorphosis and seasonality but not by pathogen infection
Source: Anim Microbiome. 2022 Dec 12;4:63. doi: 10.1186/s42523-022-00215-7 (PMC9743558; doi:10.1186/s42523-022-00215-7)
Supplement: Supplementary file 1 — Additional file 1. Supplementary Figures. [file 42523_2022_215_MOESM1_ESM.docx]

**Supplementary Figures:**

**The skin microbiota of the axolotl *Ambystoma altamirani* is highly influenced by metamorphosis and seasonality but not by pathogen infection.**

**Emanuel Martínez-Ugalde^1^, Víctor D. Ávila-Akerberg^2^, Tanya M. González Martínez^3^, Montserrat Vázquez Trejo^3^, Dalia Zavala Hernández^3^, Sara Anaya Morales^1^, Eria A. Rebollar^1^.**

1 Centro de Ciencias Genómicas, Universidad Nacional Autónoma de México

2 Instituto de Ciencias Agropecuarias y Rurales, Universidad Autónoma del Estado de México

3 Facultad de Ciencias, Universidad Nacional Autónoma de México

**Figure S1.** Taxonomic composition of *A. altamirani* skin samples and environmental samples: Metamorphic (M), pre-metamorphic (PM), sediment (S) and water samples (W). Stacked bar plots show the average relative abundances of the ten most abundant bacterial families. Sample size is shown below each bar.

**Figure S2.** *A. altamirani* skin bacterial alpha and beta diversity across sampling locations. A) Phylogenetic diversity (PD) in metamorphic samples. B) Principal coordinate analysis (PCoA) based on weighted Unifrac distances for metamorphic samples C) PD for pre-metamorphic samples. D) Principal coordinate analysis (PCoA) based on weighted Unifrac distances for pre-metamorphic samples. Circles are color-coded by sampling site.

**Figure S3.** Kendall significant correlations between Bd infection loads and bacterial relative abundance in infected samples. A) Correlated ASVs on metamorphic samples. B) Correlated ASVs on pre-metamorphic samples. Y axis shows Kendall τ values for significant correlations, X axis shows the relative abundance of each ASV. Circles are color-coded by bacterial phylum
