# Supplementary material for: The skin microbiota of the axolotl Ambystoma altamirani is highly influenced by metamorphosis and seasonality but not by pathogen infection
Source: Anim Microbiome. 2022 Dec 12;4:63. doi: 10.1186/s42523-022-00215-7 (PMC9743558; doi:10.1186/s42523-022-00215-7)
Supplement: Supplementary file 2 — Additional file 2. Supplementary Tables. [file 42523_2022_215_MOESM2_ESM.docx]

**Supplementary Tables**

**The skin microbiota of the axolotl *Ambystoma altamirani* is highly influenced by metamorphosis and seasonality but not by pathogen infection.**

**Emanuel Martínez-Ugalde^1^, Víctor D. Ávila-Akerberg^2^, Tanya M. González Martínez^3^, Montserrat Vázquez Trejo^3^, Dalia Zavala Hernández^3^, Sara Anaya Morales^1^, Eria A. Rebollar^1^.**

1 Centro de Ciencias Genómicas, Universidad Nacional Autónoma de México

2 Instituto de Ciencias Agropecuarias y Rurales, Universidad Autónoma del Estado de México

3 Facultad de Ciencias, Universidad Nacional Autónoma de México

**Table S1.** *Post-hoc* pairwise Wilcoxon tests for Shannon, observed ASV and Phylogenetic diversity (PD). Numbers in bold indicate significant p-values of paired comparisons. Metamorphic (M), pre-metamorphic (PM), sediment (S), water (W).

|  | Shannon | Observed ASVs | PD |
| --- | --- | --- | --- |
| M-PM | **7.20e^-21^** | **9.44e^-20^** | **4.95e^-19^** |
| M-S | **6.80e^-25^** | **2.20e^-23^** | **8.32e^-26^** |
| M-W | 0.66 | 0.48 | **0.011** |
| PM-S | **1.10e^-36^** | **2.52e^-36^** | **2.06e^-36^** |
| PM-W | **1.00e^-20^** | **3.29e^-24^** | **2.92e^-26^** |
| S-W | **3.30e^-25^** | **8.00e^-25^** | **6.80e^-25^** |

**Table S2.** Pairwise PERMANOVA comparisons between sample types. Numbers in **bold** indicate statistically significant p-values of each comparison.

| Group 1 | Group 2 | Sample size | Permutations | pseudo-F | p-value | q-value |
| --- | --- | --- | --- | --- | --- | --- |
| Metamorphic | Pre-metamorphic | 272 | 999 | 50.20 | **0.001** | **0.0012** |
| Metamorphic | Sediment | 159 | 999 | 87.20 | **0.001** | **0.0012** |
| Metamorphic | Water | 156 | 999 | 4.32 | **0.002** | **0.002** |
| Pre-metamorphic | Sediment | 267 | 999 | 110.51 | **0.001** | **0.0012** |
| Pre-metamorphic | Water | 264 | 999 | 42.40 | **0.001** | **0.0012** |
| Sediment | Water | 151 | 999 | 111.25 | **0.001** | **0.0012** |

**Table S3.** Analysis of multivariate homogeneity of group dispersions between sample types. Numbers above the diagonal represent the permuted p-values while numbers bellow the diagonal represent observed p-values. Numbers in **bold** indicate significant p-values.

|  | Metamorphic | Pre-metamorphic | Sediment | Water |
| --- | --- | --- | --- | --- |
| Metamorphic |  | **1.00e^-03^** | **1.00e^-03^** | 0.269 |
| Pre-metamorphic | **8.96e^-06^** |  | **1.00e^-03^** | **0.001** |
| Sediment | **1.07e-^06^** | **5.33e^-18^** |  | **0.002** |
| Water | 0.24 | **9.05e^-08^** | **1.07e^-03^** |  |

**Table S4.** Amplicon sequence variants (ASVs) defining the bacterial core of sediment and water samples. ASVs that are shared with the metamorphic bacterial core are highlighted in **bold**.

|  | ASV ID | Taxonomy at family level | Relative abundance | Persistence |
| --- | --- | --- | --- | --- |
| Sediment | **6d0c9d0395e6a2a7667eb0b07c17a275** | **Burkholderiaceae** | **2.32** | **100.00** |
|  | 5092297d795976b29921946d1d7aff2f | Spirosomaceae | 0.68 | 97.40 |
|  | 8fd9eab61a0f63db6cbb201ce66b484b | Burkholderiaceae | 0.78 | 100.00 |
|  | 4eece736dad7d5a26e82e2d2247d3a9b | Burkholderiaceae | 0.67 | 98.70 |
|  | f5944006a2164242b3d35b6f35269be2 | Burkholderiaceae | 0.60 | 97.40 |
|  | 6725ac4b1fde87ad69cedaf457fd2376 | Burkholderiaceae | 0.55 | 96.10 |
|  | 3b20703d6681b21538748d73a9b83dc0 | Pedosphaeraceae | 0.57 | 94.81 |
|  | 0124b03431ca1882134f36a8cb86c9b9 | Pedosphaeraceae | 0.55 | 98.70 |
|  | ac415ad279cb27c6caecc356aef37309 | Pedosphaeraceae | 0.45 | 96.10 |
|  | 6edfdf296fc994a55f9981dda43d242a | Rhodobacteraceae | 0.44 | 97.40 |
|  | 282ecd235de20ec5393575e4e27582f7 | Xanthobacteraceae | 0.44 | 94.81 |
|  | 5cb5f60773d589f49624fb3e212416d0 | Pedosphaeraceae | 0.38 | 98.70 |
|  | dc3ea98362f99244fce2ece1cc293384 | Geobacteraceae | 0.42 | 98.70 |
|  | 74aa1a6159865a8305e5fd7e98901154 | Burkholderiaceae | 0.36 | 93.51 |
|  | 91291567e47efece2472186165552086 | Nitrosomonadaceae | 0.34 | 98.70 |
|  | 89e24b9cf9c0922557315bebd4765fca | Chthoniobacteraceae | 0.27 | 100.00 |
|  | 8da392b60860c28c114f4d1696bcb918 | Microscillaceae | 0.27 | 93.51 |
|  | 69be5205c4f39f10d94806fd7abd891c | Microscillaceae | 0.21 | 93.51 |
|  | 926a77485ba849b0dcb6f0b0fa54864b | TRA3-20 | 0.24 | 92.21 |
|  | 841890b0612bcb02e31dab6907df8d1d | Cellvibrionaceae | 0.26 | 97.40 |
|  | 64f6423765becb7998eb54f0bb4a514f | uncultured proteobacterium | 0.18 | 90.91 |
|  | e2022b2ee4476a7c92a637dcd9887deb | env.OPS 17 | 0.18 | 100.00 |
|  | 852faa226ea1900e5befddd8f336e71e | Chitinophagaceae | 0.17 | 92.21 |
|  | 8462829dec031a181482fc4c0fb1481c | Opitutaceae | 0.16 | 92.21 |
|  | d07e75050a4e4029595a126ae8683d50 | Burkholderiaceae | 0.16 | 98.70 |
|  | 0ccbe75e33f75230126df77dfaf8f511 | Devosiaceae | 0.15 | 94.81 |
|  | 8813fd04fb20de3070defbb994e31ebb | metagenome | 0.13 | 90.91 |
|  | 1e74e07ca37faaf31b1c59378c54d654 | Chitinophagaceae | 0.10 | 90.91 |
|  | 4150e5d078d1f5e24dd501083242f7bf | uncultured Holophaga sp. | 0.10 | 92.21 |
|  | 19373c69bd87f24161bd8fb593a988ef | P3OB-42 | 0.07 | 96.10 |
| Water | **9936daae333af6e517a9deb4b9e18ffa** | **Pseudomonadaceae** | **17.19** | **100.00** |
|  | **be8eb25874b4202cf98050dbadeeb7ce** | **Burkholderiaceae** | **0.31** | **94.59** |

**Table S5.** Two-way Multivariate Analysis of Variance evaluating physicochemical variation across seasons and sampling locations. Mean temp (mean temperature), Max temp (maximum temperature), Min temp (minimal temperature), Delta temp (difference between max and min temp), DO (dissolved oxygen), Cond (conductivity). Numbers in bold indicate significant p-values.

|  | Season | | | | | Locality | | | | |
| --- | --- | --- | --- | --- | --- | --- | --- | --- | --- | --- |
|  | Df | Sum Sq | Mean Sq | F-value | p-value | Df | Sum Sq | Mean Sq | F-value | p-value |
| Mean temp | 3 | 71.37 | 23.79 | 103.85 | **2.20e^-16^** | 3 | 48 | 15.99 | 69.83 | **3.68e^-16^** |
| Max temp | 3 | 68.61 | 22.87 | 24.24 | **3.47e^-09^** | 3 | 159.54 | 53.18 | 56.38 | **1.32e^-14^** |
| Min temp | 3 | 35.31 | 11.77 | 12.40 | **6.53e^-06^** | 3 | 81.69 | 27.23 | 28.69 | **3.66e^-10^** |
| Delta temp | 3 | 134.94 | 44.979 | 14.68 | **1.22e^-06^** | 3 | 466.09 | 155.36 | 50.7 | **7.41e^-14^** |
| pH | 3 | 2.55 | 0.85 | 14.85 | **1.08e^-06^** | 3 | 3.005 | 1.001 | 17.48 | **1.83e^-07^** |
| DO | 3 | 5429 | 1809.65 | 66.16 | **9.20e^-16^** | 3 | 83.8 | 27.92 | 1.02 | 0.3933 |
| Cond | 3 | 1315.2 | 438.4 | 3.538 | **0.022** | 3 | 24675.9 | 8225.3 | 66.38 | **8.69e^-16^** |

**Table S6.** *Post-hoc* pairwise Wilcoxon tests of Phylogenetic diversity (PD) for metamorphic and pre-metamorphic axolotls across seasons. Numbers in bold indicate significant p-values of paired comparisons between consecutive seasons. Summer (S), autumn (Aut), winter (Win), spring (Spr).

|  | Metamorphic | Pre-metamorphic |
| --- | --- | --- |
| Sum-Aut | 0.801 | 0.93 |
| Sum-Win | 0.451 | 0.93 |
| Sum-Spr | 0.009 | 0.93 |
| Aut-Win | 0.382 | 0.93 |
| Aut-Spr | 0.019 | 0.93 |
| Win-Spr | **0.005** | 0.93 |

**Table S7.** Pairwise PERMANOVA pairwise comparisons between seasons for metamorphic samples. Numbers in **bold** represent statistically significant p-values between consecutive seasons.

| Group 1 | Group 2 | Sample size | Permutations | pseudo-F | p-value | q-value |
| --- | --- | --- | --- | --- | --- | --- |
| Autumn | Spring | 48 | 999 | 22.4070207 | 0.001 | 0.002 |
| Autumn | Summer | 51 | 999 | 1.2168406 | 0.302 | 0.302 |
| Autumn | Winter | 35 | 999 | 1.39273164 | 0.217 | 0.302 |
| Spring | Summer | 47 | 999 | 21.4223451 | 0.001 | 0.002 |
| Spring | Winter | 31 | 999 | 14.9815017 | **0.001** | **0.002** |
| Summer | Winter | 34 | 999 | 1.2181662 | 0.292 | 0.302 |

**Table S8.** Analysis of multivariate homogeneity of group dispersions between seasons for metamorphic axolotls. Numbers above the diagonal represent the permuted p-values while numbers bellow the diagonal represent observed p-values. Numbers in **bold** indicate significant p-values.

|  | Summer | Autumn | Winter | Spring |
| --- | --- | --- | --- | --- |
| Summer |  | 0.979 | 0.993 | 0.066 |
| Autumn | 0.980268 |  | 0.999 | 0.044 |
| Winter | 0.987862 | 0.999101 |  | 0.137 |
| Spring | 0.065539 | 0.037628 | 0.122359 |  |

**Table S9.** Pairwise PERMANOVA pairwise comparisons between seasons for pre-metamorphic samples. Numbers in **bold** represent statistically significant p-values between consecutive seasons.

| Group 1 | Group 2 | Sample size | Permutations | pseudo-F | p-value | q-value |
| --- | --- | --- | --- | --- | --- | --- |
| Autumn | Spring | 86 | 999 | 24.0061607 | 0.001 | 0.0012 |
| Autumn | Summer | 70 | 999 | 0.51792264 | 0.58 | 0.58 |
| Autumn | Winter | 92 | 999 | 13.4781367 | **0.001** | **0.0012** |
| Spring | Summer | 98 | 999 | 24.1275248 | 0.001 | 0.0012 |
| Spring | Winter | 120 | 999 | 12.6138699 | **0.001** | **0.0012** |
| Summer | Winter | 104 | 999 | 11.2951067 | 0.001 | 0.0012 |

**Table S10.** Analysis of multivariate homogeneity of group dispersions between seasons for pre-metamorphic axolotls. Numbers above the diagonal represent the permuted p-values while numbers bellow the diagonal represent observed p-values. Numbers in **bold** indicate significant p-values.

|  | Summer | Autumn | Winter | Spring |
| --- | --- | --- | --- | --- |
| Summer |  | 0.544 | 0.095 | 0.063 |
| Autumn | 0.550837 |  | 0.043 | **0.027** |
| Winter | 0.093814 | **0.036321** |  | 0.608 |
| Spring | 0.057611 | **0.029374** | 0.600254 |  |

**Table S11.** *Post-hoc* pairwise Wilcoxon tests for Phylogenetic diversity (PD) for metamorphic and pre-metamorphic axolotls between sampling locations. Numbers in bold indicate significant p-values of paired comparisons. Sampling location 1 (S1), sampling location 2 (S2), sampling location 3 (S3), sampling location 4 (S4).

|  | Metamorphic | Pre-metamorphic |
| --- | --- | --- |
| S1-S2 | 0.312 | **0.00098** |
| S1-S3 | 0.312 | 0.07816 |
| S1-S4 | 0.666 | **0.00062** |
| S2-S3 | **0.032** | 0.36012 |
| S2-S4 | 0.312 | **1.70E-07** |
| S3-S4 | 0.945 | **6.70E-05** |

**Table S12.** Pairwise PERMANOVA pairwise comparisons between sampling locations for metamorphic samples. Numbers in **bold** represent statistically significant p-values.

|  |  | Sample size | Permutations | pseudo-F | p-value | q-value |
| --- | --- | --- | --- | --- | --- | --- |
| Group 1 | Group 2 |  |  |  |  |  |
| Site 1 | Site 2 | 67 | 999 | 1.61 | 0.157 | 0.2355 |
|  | Site 3 | 23 | 999 | 2.43 | 0.039 | 0.117 |
|  | Site 4 | 14 | 999 | 0.81 | 0.504 | 0.504 |
| Site 2 | Site 3 | 68 | 999 | 5.73 | **0.002** | **0.012** |
|  | Site 4 | 59 | 999 | 1.38 | 0.255 | 0.306 |
| Site 3 | Site 4 | 15 | 999 | 1.75 | 0.121 | 0.2355 |

**Table S13.** Pairwise PERMANOVA pairwise comparisons between sampling locations for pre-metamorphic samples. Numbers in bold represent statistically significant p-values.

|  |  | Sample size | Permutations | pseudo-F | p-value | q-value |
| --- | --- | --- | --- | --- | --- | --- |
| Group 1 | Group 2 |  |  |  |  |  |
| Site 1 | Site 2 | 101 | 999 | 57.7 | **0.001** | **0.0015** |
|  | Site 3 | 89 | 999 | 3.26 | **0.041** | **0.041** |
|  | Site 4 | 118 | 999 | 9.2 | **0.001** | **0.0015** |
| Site 2 | Site 3 | 72 | 999 | 60.44 | **0.001** | **0.0015** |
|  | Site 4 | 101 | 999 | 88.37 | **0.001** | **0.0015** |
| Site 3 | Site 4 | 89 | 999 | 4.97 | **0.005** | **0.006** |

**Table S14.** Analysis of multivariate homogeneity of group dispersions between sampling locations for metamorphic axolotls. Numbers above the diagonal represent the permuted p-values while numbers bellow the diagonal represent observed p-values. Numbers in **bold** indicate significant p-values.

|  | S1 | S2 | S3 | S4 |
| --- | --- | --- | --- | --- |
| S1 |  | 0.45 | 0.411 | 0.988 |
| S2 | 0.46433 |  | 0.902 | 0.661 |
| S3 | 0.42941 | 0.88271 |  | 0.244 |
| S4 | 0.98528 | 0.65149 | 0.24319 |  |

**Table S15.** Analysis of multivariate homogeneity of group dispersions between sampling locations for pre-metamorphic axolotls. Numbers above the diagonal represent the permuted p-values while numbers bellow the diagonal represent observed p-values. Numbers in **bold** indicate significant p-values.

|  | S1 | S2 | S3 | S4 |
| --- | --- | --- | --- | --- |
| S1 |  | 0.08 | **6.00E-03** | 0.095 |
| S2 | 0.09 |  | **1.00E-03** | **0.003** |
| S3 | **6.54E-03** | **6.72E-05** |  | 0.134 |
| S4 | 0.11 | **1.47E-03** | 0.12 |  |

**Table S16**. Variance explained of each canonical axis calculated by dbRDA. Columns indicate: F statistic, p-values, variance explained by each canonical axis, and the cumulative variance calculated by the Permutational like ANOVA. Numbers in **bold** indicate significant p-values and the cumulative variance for each statistically significant canonical axis.

|  | F | p-value | Variance explained | Cumulative variance |
| --- | --- | --- | --- | --- |
| CAP1 | 63.3875 | **0.001** | **0.173** | **0.173** |
| CAP2 | 18.4679 | **0.001** | **0.050** | **0.223** |
| CAP3 | 9.8065 | **0.001** | **0.026** | **0.250** |
| CAP4 | 5.1977 | **0.006** | **0.014** | **0.264** |
| CAP5 | 2.5146 | 0.123 | 0.006 | 0.271 |
| CAP6 | 1.5675 | 0.387 | 0.004 | 0.275 |
| CAP7 | 1.1402 | 0.563 | 0.003 | 0.279 |
| CAP8 | 0.7233 | 0.764 | 0.001 | 0.281 |

**Table S17.** ASVs significantly correlated with Bd infection intensity and that are present in both metamorphic and pre-metamorphic samples. ASVs which differ in the type of correlation (positive or negative) between sample types (metamorphic/pre-metamorphic) are highlighted in **bold**.

| OTU | Metamorphic p-value | Metamorphic Kendall τ | Pre-metamorphic p-value | Pre-metamorphic Kendall τ | Best hit |
| --- | --- | --- | --- | --- | --- |
| 6b9a5f7e85b05d17e49402afedb5d09d | 0.0062 | 0.30667 | 0.0025 | 0.2083 | Sphingobacteriaceae (F) |
| 4a05a9e45659d8b2597fd39eccebcc69 | 0.0150 | 0.2602 | 0.0011 | 0.2068 | Flavobacteriaceae (F) |
| d42988b9162532f845c1fb5f283e0e36 | 0.04237 | 0.2309 | 0.0213 | 0.1594 | Opitutaceae (F) |
| f5343248f66d395a4a3f81f0ec2e6139 | 0.02698 | **-0.2504** | 0.0251 | **0.1553** | **Acetobacteraceae (F)** |
| bc1aac689cb18f362a259d258c62d6c3 | 0.00383 | 0.3214 | 0.0296 | 0.1498 | Saprospiraceae (F) |
| 5e9e03d7bb698e92ce2c344e381f05cc | 0.04237 | 0.2309 | 0.0314 | 0.1492 | Nitrosomonadaceae (F) |
| 0db5c0f9a31014f597a82c40a97e5047 | 0.03373 | 0.2415 | 0.0482 | 0.1370 | AKYH767 (F) |
| 3c28f0caf9183357de05d1882a943f8e | 0.02110 | **0.2242** | 0.0403 | **-0.1170** | **Chitinophagaceae (F)** |
| d2a68e0d41ffc5747e42a888d8314c92 | 0.00895 | **0.2786** | 0.0488 | **-0.1342** | **Gemmataceae (F)** |
| ee3f91c192699d4bc52b1c12179e2b00 | 0.01548 | **0.2690** | 0.0202 | **-0.1590** | **Acidobacteria (P)** |
| ec7d28b8c8ab3ddb201d48b2ad5ee90a | 0.03004 | **0.2422** | 0.0169 | **-0.1651** | **Flavobacteriaceae (F)** |
| 2ebb0a4d8c3eb5dfc6e277e14e5701e7 | 0.01223 | **0.2798** | 0.006 | **-0.1881** | **Chitinophagaceae (F)** |

**Table S18.** List of axolotl samples collected at each sampling location. Number of samples from the skin of *A. altamirani* individuals: metamorphic (M) and pre-metamorphic (PM).

|  | Summer | | Autumn | | Winter | | Spring | |  |
| --- | --- | --- | --- | --- | --- | --- | --- | --- | --- |
|  | PM | M | PM | M | PM | M | PM | M | **Tota**l |
| Site 1 | 13 | 3 | 15 | 6 | 17 | 0 | 15 | 3 | 72 |
| Site 2 | 12 | 15 | 8 | 17 | 13 | 9 | 10 | 17 | 101 |
| Site 3 | 3 | 6 | 2 | 4 | 13 | 0 | 14 | 2 | 44 |
| Site 4 | 13 | 1 | 4 | 1 | 23 | 0 | 19 | 1 | 62 |
| Total | 41 | 25 | 29 | 28 | 66 | 9 | 58 | 23 | 279 |
